# Special Considerations for Management of Diabetes in Adult Patients with Intellectual and Developmental Disabilities

**DOI:** 10.1155/2023/2955772

**Published:** 2023-01-30

**Authors:** Rebecca Oyetoro, Layton Wiemer, Olgert Bardhi, Mariam Louis, Rafik Jacob

**Affiliations:** ^1^University of Florida, Gainesville, FL, USA; ^2^University of Florida, Jacksonville, FL, USA

## Abstract

Diabetes mellitus (DM) is a chronic health condition that is very prevalent worldwide. It has been demonstrated that individuals with intellectual and developmental disabilities (IDDs) are at a disproportionately high risk for developing diabetes. Persons with IDDs are estimated to be 2-3 times more likely to develop DM compared to the general population. The elevated risk of developing diabetes within the population of adults with IDDs is multifactorial and includes contributions from genetics, lifestyle, medication use and misuse, boundaries to appropriate medical care, a higher incidence of comorbid mental health disorders, and others. Further, inadequate screening for and management of diabetes for these patients results in heightened risk for adverse cardiovascular events and inferior health care outcomes. To improve patient outcomes for this unique patient population, health care providers need to be well trained in the optimal modalities of screening, diagnosis, and management of diabetes in adults with IDDs. This requires the development of effective diabetes intervention and health promotion programs aimed at patients with IDDs, utilizing a patient-centered approach to screening and management, and conducting further research to assess the impact of these interventions.

## 1. Introduction

About 7.4 million people in the United States (US) live with an intellectual or developmental disability [1]. Intellectual and developmental disabilities (IDDs) describe incurable, life-long disorders that disrupt the trajectories of developmental milestones. These disorders typically present at birth or have an onset before the age of 22 years [2]. The term IDDs is used to categorize a group of neurodevelopmental conditions that demonstrate impairments in cognition, communication, mobility, self-care, independent living, and adaptive behavior [3, 4]. Studies have shown that persons with IDDs are more likely to have substandard health outcomes related to less access to sufficient health care services, increased polypharmacy, higher poverty rates, inadequate nutrition, and longer periods of no physical activity [5–7].

Persons with IDDs are at greater risk for chronic health diseases including cardiovascular disease, obesity, diabetes mellitus (DM), epilepsy, constipation, kidney disease, osteoarticular disorder, and thyroid disorder [3, 8, 9]. Since most IDDs have no cure, healthcare providers (HCPs) management of this patient population largely focuses on treating symptoms and comorbidities that will result in a heightened quality of life. Due to increased access to appropriate medical intervention and preventative care, the average lifespan of persons with IDDs now extends into the later decades of life, with most people living in the range of 50–60 years of age [4–10].

There is a notable rise in the early onset of type 2 diabetes mellitus in the aging population. According to the American Diabetes Association (ADA), over 11.3% of the US population has been diagnosed with diabetes. Of the 37.3 million adults with diabetes, 8.5 million are undiagnosed [11]. Adults with IDDs have been reported to have a disproportionately high risk of developing diabetes. It is estimated that up to 16.3% of adults with IDDs have diabetes, compared with only 7.2% of their counterparts without IDDs [12]. Advances in health care have increased the life expectancy of persons with IDDs. Consequently, the prevalence of chronic health conditions such as diabetes has also increased in this patient population. There is currently insufficient evidence on health disparities for adults with IDDs. Continuum surveillance on ample health care screening, adequate nutrition, and physical activities can have a significant impact on the prevention of chronic health conditions and improving the quality of life for the aging population of individuals with IDDs [8, 9, 13, 14].

## 2. Diabetes and IDDs

Diabetes mellitus encompasses variable disorders of carbohydrate metabolism, resulting in the unifying outcome of hyperglycemia. It can be stratified into two major types—type 1 diabetes mellitus (T1DM) and type 2 diabetes mellitus (T2DM). T1DM is characterized by an absolute impairment in insulin secretion, while T2DM is described by peripheral resistance to the action of insulin with varying degrees of impairment in insulin secretion [15]. Among the US diabetic population, approximately 90% are diagnosed as having T2DM [16, 17].

Insulin plays a pivotal role in both the glucose transport pathway and glucose metabolism. It functions by binding and then activating the insulin receptor by phosphorylating key tyrosine residues on the beta chain. Defective insulin signaling leads to insulin acting as a potent growth factor which results in vasodilation, atherogenesis, and cardiometabolic disturbances. The transcription of this growth factor results in the activation of inflammatory pathways, stimulation of vascular smooth muscle cell proliferation, and insulin resistance. Insulin resistance stimulates endothelin-1 production with increased vasoconstriction of the arterial smooth muscle cells and atherogenesis [18, 19].

Diabetes is one of the most common chronic conditions among adults with IDDs. Compared to the general population, the aging IDDs population has been reported to have a higher incidence and prevalence of DM. In the US, about 1 of 6 persons with IDDs are diagnosed with diabetes compared to only 1 of 14 people in the general population. Additionally, as persons with IDDs get older, they encounter challenges in acquiring routine preventive care screening for early detection of chronic health conditions. Currently, there is minimal to no education and/or training for HCPs and the IDD community in relation to the primary prevention of diabetes.

Preventative care measures are vital in reducing the development of diabetes complications [20]. Compared with the general population, adults with IDDs are more likely to encounter diabetic complications such as diabetic ketoacidosis (DKA), heart disease, neuropathy, nephropathy, retinopathy, stroke, or limb amputation [21, 22]. With the increasing prevalence of diabetes at an earlier age in the IDD population, more information is needed to understand health disparities and the utilization of diabetes service provisions accessible to adults with IDDs.

## 3. Predisposing Factors

Persons with IDDs are estimated to be 2-3 times more likely to develop DM compared to the general population [23]. Multiple studies have attributed the increase in the prevalence of DM among persons with IDDs to the following overarching risk factors: genetics, sedentary lifestyles, poor nutrition, medication use, and health-related issues [24]. These factors also contribute to comorbid health conditions that exacerbate living conditions and other health-related issues. Persons with IDDs are more likely to experience limited access to medical care, social isolation, and mental health conditions which may place them at an additional risk of developing DM [25]. The health inequalities surrounding persons with IDDs warrant further evaluation and consideration for HCPs, caregivers, and policymakers to ensure proper steps are taken to identify needs and achieve proper care.

Although significant progress has been made in defining genetic risks for specific subtypes of diabetes such as type 1 diabetes mellitus and maturity-onset diabetes of the young, there remains much to be learned about genotypic contributions to the development of diabetes mellitus. As it pertains to persons with IDDs, we do have evidence to support that having comorbid neurodevelopmental disorders in and of itself is associated with poor glycemic control and a higher risk of diabetic complications [26]. Screening for these disorders early in childhood in the appropriate patient population can help identify disorders that strongly correlate with the risk of developing DM. Furthermore, due to having a higher propensity to experience higher levels of obesity, individuals with IDD-associated chromosomal and nonchromosomal syndromes such as down syndrome, Prader–Willi syndrome, Angelman syndrome, Turner syndrome, and Klinefelter syndrome have an increased risk of developing DM [8, 23–25].

Most primary care physicians are familiar with the two most common genetic causes of intellectual disabilities, down syndrome, and Fragile X syndrome, as these were both discovered many decades ago and are well described. However, the discovery of new syndromes associated with IDDs and the emergence of large-scale genome sequencing projects over recent years is likely to alter the way that we search for genetic anomalies in persons with IDDs in years to come. For instance, new genetic methods have been developed in recent years including microarray-based comparative genomic hybridization and whole genome or exome sequencing that can detect genetic abnormalities associated with IDDs that could not previously have been seen by conventional G-banded karyotyping. Advances like this can potentially expand the size of the group of individuals with IDDs for whom we should be vigilant about screening for the comorbid development of diabetes mellitus and the metabolic syndrome [27].

Individuals with IDDs are also disproportionately overrepresented among the population with financial insecurity [28, 29]. While the Americans with Disabilities Act (ADA) is in place to help facilitate equal opportunities for both employment and education for persons with IDDs, there remains a low labor force participation rate for adults with IDDs when compared with people without disabilities of the same age and geographic location [30]. Numerous studies have demonstrated that low socioeconomic status correlates with the likelihood of developing chronic health conditions including diabetes mellitus [31]. It has been theorized that this correlation exists because individuals with IDDs experience increased barriers to obtaining healthcare for many reasons including mobility impairments, transportation barriers, and communication difficulties. Without people with IDDs having unobstructed access to primary care physicians to help educate and implement strategies to reduce the risk of the development of DM, this trend is likely to continue.

As adults with IDDs age, there is a higher prevalence of psychiatric comorbidity [32]. According to an analysis of the US 2018 behavioral risk factor surveillance system (BRFSS), the prevalence of reported mental distress among those with disabilities (32.9%) was 4.6 times that of those without disabilities (7.2%) [12]. Unfortunately, the presence of mental health problems often goes undetected in persons with IDDs in clinical settings. These conditions include psychosis disorders, mood disorders, anxiety disorders, substance abuse disorders, and dementia. Sources of challenging behavioral disorders can be improper use/dosing of antipsychotic drugs, unrecognized physical health problems that cause pain, and polypharmacy [33]. As is true with the general population, challenging behavioral disorders are often managed with antipsychotic medications in individuals with IDDs. Antipsychotic drugs are used to treat a wide variety of behavioral disorders including but not limited to schizophrenia, bipolar disorders, acute psychosis, major depressive disorders with psychotic features, obsessive-compulsive disorders (OCD), Tourette syndrome, anxiety disorders, and Huntington's disease [34–36]. Physicians must develop an understanding of the genetic risk factors and comorbid health conditions that relate to IDDs that are associated with commonly encountered behavioral disorders.

Antipsychotic drugs are used to reduce psychotic symptoms. They are generally more effective at managing positive symptoms of psychotic disorders such as hallucinations, delusions, and disorganized thoughts and speech as compared with negative symptoms such as blunted affect and avolition. There are two classes of antipsychotic drugs, termed first-generation antipsychotics (FGAs) and second-generation antipsychotics (SGAs). FGAs exert their effects by working as dopamine receptor antagonists, while the mechanism of SGAs involves antagonistic activity at serotonin-dopamine receptors, alpha-adrenergic receptors, and antihistamine receptors. SGAs, also known as atypical antipsychotics, are often preferred in the treatment of behavioral challenges due to the reduced risk of extrapyramidal symptoms (EPS) such as dystonia, parkinsonism, and akathisia as compared with FGAs [37]. However, the risk of EPS with FGAs must be weighed against the risk of the less favorable metabolic profile that accompanies SGAs [38, 39]. SGAs, especially clozapine, olanzapine, and quetiapine are commonly associated with the development of the metabolic syndrome [40].

The metabolic syndrome is a cluster of metabolic disturbances that increase cardiometabolic events. The diagnostic criteria for metabolic syndrome are shown in Table 1. The presence of the metabolic syndrome significantly increases the risk of cardiovascular disease, obesity, insulin resistance, dyslipidemia, hyperglycemia, and hypertension [39, 40]. According to the Center for Disease Control and Prevention (CDC), between the years 1988–1994 and 2007–2012, the prevalence of metabolic syndrome increased from 25.3% to 34.2% of US adults [41]. Individuals with metabolic syndrome are five times more likely to develop T2DM [42].

The American Psychiatric Association Practice Guideline recommends that antipsychotic drugs be avoided as a first-line treatment for patients with an increased risk of obesity [43]. As previously mentioned, individuals with IDD-associated chromosomal and nonchromosomal syndromes such as down syndrome, Prader–Willi syndrome, Angelman syndrome, Turner syndrome, and Klinefelter syndrome have an increased propensity for being overweight [23–25].

Obesity, specifically visceral adiposity, is strongly associated with the development of hyperglycemia, hypertension, atherosclerosis, T2DM, and other cardiovascular diseases. In obese individuals, the excess accumulation of fat in adipocytes releases proinflammatory cytokines that inhibit the secretion of adiponectin (an insulin-secreting molecule) [44, 45]. Insulin resistance has been demonstrated to be a strong predictor of atherosclerotic cardiovascular disease (ASCVD). Insulin resistance also develops due to lipotoxicity, the elevation of plasma nonesterified fatty acids, altered fat topography, and adiposopathy [45–47]. However, the hyperglycemic-related adverse effects are not well understood. Additionally, multiple studies have shown a correlation between hyperglycemia and ketoacidosis in patients treated with antipsychotic drugs [48].

The aforementioned highlights the critical importance of HCPs optimizing nonpharmacologic means of therapy before initiating medications with potentially harmful adverse effect profiles. This should include a multidisciplinary approach to patient care that promotes healthy lifestyle recommendations such as proper nutrition and regular physical activity, provides appropriate access to mental health screenings, encourages community participation, and takes quality of life measures into account [43]. When prescribing antipsychotic drugs to persons with IDDs, HCPs should confirm the diagnosis of mental illness to determine whether medical therapy or lifestyle modification is warranted. Next, an individualized treatment plan is needed to routinely assess comorbidities, communication barriers, the complexity of care, and screening for cardiometabolic risk [35].  Table 2 highlights some of the special considerations for providers managing adults with IDDs. Persons with IDDs and their caregivers should also be given a detailed education about the antipsychotic drug and its potential adverse effects. Medications should be started with a low dose, and dosing should be increased vigilantly [34–37, 43].

## 4. Improving Care for Persons with IDDs

Persons with IDDs have been recommended as an important group to consider for diabetes screening and prevention strategies, given their higher risk of developing T2DM. This is in part due to diabetes management guidelines not being met because of a lack of regular screening of blood glucose levels, body mass index, cholesterol, and blood pressure. A 2007 study explored the correlation between BMI and cardiometabolic risk factors using two large surveys nationally representative of the US adult population, the SHIELD 2004 screening questionnaire and the NHANES 1999–2002. The study showed a significant (*p* < 0.001) increase in the prevalence of DM associated with increased BMI in both survey groups [24]. To date, there have been very few studies that investigate or evaluate diabetes management and prevention specifically in persons with IDDs, none of which have been done within the US despite an increasing population of people with diabetes.

Nonetheless, the programs that do exist outside of the U.S. offer a glimpse of how to achieve successful diabetes health promotion and prevention in individuals with IDDs. In the United Kingdom, there is one diabetes prevention program (STOP Diabetes) [49, 50] and two self-management T2DM education programs (DESMOND-ID; OK diabetes) [51, 52] adapted for patients with IDDs. Each of the programs targets lifestyle factors such as weight management for weight-reduction strategies to reduce the risk of developing T2DM and cardiovascular disease. These programs are tailored specifically to participants and family members to achieve reasonable adjustments while preserving patient autonomy.

Diabetes is typically identified and diagnosed by HCPs in patients with certain risk factors. Given that patients with IDDs are known to be at elevated risk for developing T2DM, HCPs should have a low threshold for screening this patient population for signs and symptoms of hyperglycemia. For example, persons with IDDs should be asked about symptoms of polydipsia, polyuria, polyphagia, and weakness. Patients who endorse signs or symptoms of hyperglycemia should be screened for T2DM and monitored at each routine visit. Preventative measures such as timely screening and proper follow-up must be incorporated into inpatient visits routinely. HCPs are at the frontline of direct patient care; therefore, it is critical that increased training and educational opportunities specifically aimed at taking care of comorbid conditions in patients with IDDs be made available [43, 53, 54]. Once a diagnosis of T2DM is made in an individual with or without IDDs, a primary treatment goal for optimal outcomes for most people with diabetes mellitus is reaching a target HbA1c of <7% [21].

The United States Preventive Services Task Force (USPSTF) recommends offering or referring adults who are overweight or obese and have additional cardiovascular disease (CVD) risk factors (hypertension, dyslipidemia, impaired blood sugar, or metabolic syndrome) to intensive behavioral counseling interventions to promote a healthful diet and physical activity for cardiovascular disease prevention. The USPSTF also recommends screening for T2DM in adults aged 35–70 years who are overweight or obese [55]. The primary approach in diabetes management is implementing intensive behavioral counseling interventions to improve glycemic outcomes. Evidence has shown a reduction in mortality rates in diabetes management following the implementation of healthy dietary habits and weight reduction. The consumption of fruits and vegetables and a low carbohydrate, salt, cholesterol, saturated fat, and trans-fat diet has been found to be an effective metabolic intervention [55–59]. The United Stated Food and Drug Administration (US FDA) recommends 2000 kcal/day for an average person. Individuals with increased cardiometabolic risk should limit their caloric intake to 1200–1500 kcal/day in women and 1500–1800 kcal/day in men to achieve weight loss [59]. Weight loss between 5–10% has been shown to improve the sensitivity of insulin resistance and reduce the risk of metabolic syndrome. Other lifestyle interventions include regular physical activity (moderate aerobic activity of at least 30 minutes, 5–7 times a week), improving blood pressure, moderate consumption of alcohol, and smoking cessation [60].

Further, proper diabetes management requires that medications be taken as recommended by established guidelines. This presents a challenge for physicians managing patient's with diabetes because it is well known that poor medication-taking is not an uncommon occurrence in persons with diabetes. One recent study compared the frequency and factors associated with diabetes medication-taking in people with mild to moderate intellectual disabilities and those without intellectual disability. This study found that while there was no statistically significant difference in the frequency of medication-taking amongst the two groups, people with intellectual disabilities and diabetes had significantly higher levels of perceived side effects from their diabetes medications [61]. These results highlight the importance of increasing pharmacovigilance and screening for side effects by family members, caregivers, and HCPs of individuals with IDDs receiving pharmacologic therapy for DM. The flowchart within Table 3 provides a model which can be used to help simplify the comprehensive management of T2DM into its core elements.

## 5. Treatment of T2DM for Persons with IDDs

In general, the pharmacologic treatment of T2DM in adult patients with IDDs should mirror the management strategies used for treatment of T2DM in patients without IDDs. However, as discussed throughout this review, individuals with IDDs have unique risk factors, healthcare disparities, and medical needs that require increased education and modification to management plans on the part of the clinician. The existing literature fails to provide clear recommendations on how to best optimize T2DM treatment plans for adults with IDDs. We will briefly review the profiles of the pharmacologic agents routinely used in guideline-directed management of T2DM in adults and then subsequently analyze how they play into optimizing management for patients with IDDs.

Antidiabetic agents are shown to be effective in lowering blood glucose and hemoglobin A1c. The first-line therapy for glucose-lowering medication for individuals with T2DM is generally metformin. Metformin is in the biguanides drug class and works by increasing peripheral insulin sensitivity and hepatic glucose uptake. Beyond its antihyperglycemic properties, it has also demonstrated a reduction of LDL-C levels and an elevation in HDL-C levels. Emerging studies have shown metformin-related weight loss; however, the mechanism is unclear [62]. It is notable that this medication class is contraindicated in patients with an eGFR less than 30 mL/min/1.73 m^2^ or with comorbid conditions otherwise predisposing to lactic acidosis.

The insulin-sensitizing antidiabetic agents, thiazolidinediones (TZDs), have proven to be an effective medication class in individuals with metabolic syndrome. TZDs act by activating peroxisome proliferator-activated receptors (PPAR*γ*) and regulate adipogenesis to enhance intracellular fat oxidation by adipose remodeling. PPAR*γ* lowers obesity-related metabolic complications with the reduction of adipose accumulation in visceral depots. TZDs have demonstrated an improved insulin sensitivity by reducing proinflammatory pathways and inhibiting atherogenesis that is associated with the development of insulin resistance [63]. In the PERISCOPE trial, pioglitazone was shown to lower coronary atherosclerotic plaque volume in the arterial vasculature. In the PROactive study, it was suggested that patients with T2DM and a prior cardiovascular event who used pioglitazone therapy had a reduction in cardiovascular mortality by 16%. Despite the observed reduction in cardiovascular mortality, there was a higher risk of weight gain [64, 65]. Unfortunately, the use of TZDs is limited by the fact that they are not advised for use in patients with heart failure [66].

Another class of drugs that improves glycemic control and is frequently utilized in patients with cardiorenal comorbidities who cannot take metformin is incretins. Glucagon-like peptide 1 (GLP-1) receptor agonists work by stimulating postprandial insulin secretion endogenously and inhibit glucagon secretion by slowing gastric emptying under hyperglycemic conditions. This delayed breakdown increases satiety and slows down the breakdown of glucose from food into the bloodstream. Dipeptidyl peptidase 4 (DDP-4) inhibitors are a similar class of diabetes medication that work by increasing endogenous GLP-1. These medications are weight neutral and reduce postprandial elevation in triglycerides. Additionally, GLP-1 receptor agonists have been shown to demonstrate cardiorenal benefit [62, 67].

Sodium-glucose cotransporter 2 (SGLT2) inhibitors (empagliflozin, canagliflozin, and dapagliflozin) are also blood glucose-lowering agents with demonstrated cardiorenal benefit. These medications work by reducing renal tubular glucose reabsorption, resulting in a reduction in blood glucose. Notable adverse effects of this class of medications include an increased incidence of urinary tract infections and vaginal yeast infections, as well as euglycemic diabetic ketoacidosis [62].

The American Diabetes Association and the European Association for the Study of Diabetes released a position statement addressing the utility of antidiabetic medications for individuals with cardiometabolic risk factors [68]. Multiple studies have shown that GLP-1 receptor agonists and DDP-4 inhibitors have a more favorable safety profile. GLP-1 receptor agonists do not demonstrate negative cardiovascular effects in individuals with T2DM. For individuals with ASCVD or chronic kidney disease, sodium-glucose cotransporter-2 (SGLT-2) inhibitors can be used in combination therapy with GLP-1 and DDP-4 inhibitors for individuals with T2DM whose HbA1c is higher than the target goal. These drugs have a low risk of hypoglycemia and help promote weight loss [62].

In addition to antidiabetic agents, a multidrug regimen may be required for other comorbidities. Other medical therapies used in the treatment of cardiometabolic diseases include anti-hypertensive agents, guideline-directed medical therapy for heart failure, and lipid-lowering agents. Certain antipsychotic drugs, specifically SGAs (clozapine and olanzapine) and low-potency FGAs, have been shown to cause dyslipidemia and obesity [69]. An increase in insulin resistance has been shown to directly impact fasting triglycerides as an inappropriate level of lipolysis leads to the release of excess amounts of free fatty acids that are hepatically converted into triglycerides. Individuals should receive treatment with lipid-lowering drug agents like statins [21, 39].

Further studies have shown improvement in glycemic control with favorable lipid profiles following treatment with statins or other lipids. Statins have been shown to enhance hepatic insulin sensitivity and improve endothelial-mediated vasodilatory responses in individuals with metabolic syndrome. Statins inhibit HMG-CoA reductase which reduces plasma levels of cholesterol intracellularly by increasing hepatic cholesterol uptake through the upregulation of LDL receptors and apo B-containing lipoproteins [70]. In a clinical study assessing the effectiveness of atorvastatin in individuals with T2DM, the use of 10 mg of atorvastatin daily was found to be safe, well-tolerated, and effective in reducing LDL-C to target levels [71, 72]. In a literature review analyzing data on the efficacy of adding ezetimibe to statin therapy in ASCVD risk reduction, the ezetimibe-statin combination therapy was shown to be effective in lowering LDL-C levels when the maximal statin monotherapy regimen is unable to lower the LDL-C levels [73, 74].

When making management decisions for T2DM in individuals with IDDs, it is important to have in-depth discussions regarding treatment options, alternatives, side effects, contraindications, weight impact, expected outcomes, and routes of administration for each medication. Additionally, it is critical to simplify this information as much as is feasible and communicate it in a way that the patient and/or their caregiver can understand to help facilitate the making of an informed decision. This is particularly important in this patient population because, in many cases, it is impractical to expect people with IDDs to have the resources and a robust enough support network to have assistance in all elements of their health care. Therefore, it becomes critical to empower these individuals by educating them about how to provide reasonable degrees of self-care regarding their chronic health conditions such as diabetes.  Table 4 provides an overview of much of the information that is pertinent to include in conversations with patients when trying to make shared decisions regarding diabetes management in patients with IDDs.

The importance of involving individuals with IDDs and their caretakers in policy making and implementation and in selecting medication regimens and lifestyle modification strategies for management of their diabetes and other comorbidities cannot be overstated. Additionally, for those with IDDs who live in assisted living facilities, efforts should also be made to engage the professional staff of such facilities to stimulate and support the development of self-management skills for chronic health conditions such as diabetes in people with IDDs. For example, this can be done by providing opportunities for these patients and their caregivers to administer their own medication or check their own blood glucose rather than solely having facility staff members perform these activities.

One of the factors more pertinent to individuals with IDDs that should also always be addressed is educating them on the frequency of needlesticks required for various modalities of diabetes management. For instance, insulin administration requires needlestick glucose checks at least twice daily, if not more frequently. While strategies such as explaining what is happening in simple, direct language and utilizing relaxation and distraction techniques during needlesticks can be helpful, there are ultimately some patients for whom the anxiety around needles outweighs the benefit that these treatment modalities provide. Further, many of the diabetes medications portend a risk of hypoglycemia if patients do not take them with food, such as sulfonylureas. Patients with unpredictable diet patterns, which are seen more commonly in individuals with IDDs, are not ideal candidates for these medications. Additionally, if a person with IDDs is experiencing hypoglycemia, the presentation may be atypical and go unrecognized as these patients often have difficulty communicating their needs and are on psychiatric medications that may blunt the sympathetic response to hypoglycemia. An overarching concept in managing diabetes in adults with IDDs is that more gravity should be given to the importance of a discussion of side effect profiles when selecting medications. Individuals with IDDs often experience more side effects, have difficulty in reporting side effects, and are often underrepresented in studies in which the frequency of adverse effects for these medications was determined [61].

## 6. Conclusions

Adults with IDDs are more likely to develop T2DM compared to the general population due to genetics, lifestyle challenges, and comorbid conditions. Additionally, this patient population is more prone to unique challenges such as issues with communication, an understanding of disease processes, and increased difficulty with medication adherence. Special considerations and evaluations need to be placed on individual metabolic and dietary needs and multi-drug interactions. The goal of diabetes management for all persons diagnosed with diabetes is to prevent complications and improve quality of life [23, 43, 78].

Providers are encouraged to use a patient-centered approach that includes a comprehensive cardiovascular risk screening tool when developing treatment goals for adults with IDDs diagnosed with T2DM [55, 59]. Due to the high prevalence of behavioral challenges associated with adults with IDDs, they are more likely to be placed on antipsychotic medications. These medications have a propensity to induce cardiac and metabolic abnormalities such as obesity, dyslipidemia, and insulin resistance. In addition to routine health checks, HCPs should be familiar with various safety profiles when prescribing medications to ameliorate complications [21, 59]. The use of antihyperglycemic, antihyperlipidemic, and antihypertensive agents is beneficial in the reduction of cardiovascular events in adults with IDDs.

There have been very few diabetes intervention and health promotion programs aimed at patients with IDDs, and most of those in existence have originated outside of the US [49–53]. Given the varied health differences and diversity among patients with IDDs, health promotion programs and disease management initiatives need to be tailored to individual patients and enriched by increased patient and healthcare provider education and policy changes. Further research and program development are needed in the US to increase the well-being of people with IDDs and diabetes.

## Figures and Tables

**Table 1:** Diagnostic criteria for metabolic syndrome.

Metabolic syndrome is defined by having 3 or more of the following risk factors [40]:
Elevated waist circumference (≥102 cm in men; ≥88 cm in women)

Elevated triglycerides (≥150 mg/dL) or on drug treatment for elevated triglycerides

Elevated blood pressure (systolic ≥130 mm Hg and/or diastolic ≥85 mm Hg) or on drug treatment for elevated blood pressure

Elevated fasting glucose (≥100 mg/dL) or on drug treatment for elevated glucose

Reduced HDL cholesterol (≤40 mg/dL in men; ≤50 mg/dL in women) or on drug treatment for reduced HDL cholesterol

**Table 2:** Special considerations for providers managing adults with IDDs.

Annual health checks	(i) Encourage people with IDDs to have at least an annual health check to identify a wide range of previously unidentified conditions including diabetes mellitus

Tests	(i) Use “easy read” information which explains tests being done and how those results will affect care (ii) Many people with IDDs are fearful of needles and may need extra support and special adjustments to ensure tests take place (iii) Make sure the outcomes of any tests are communicated to the person in language that they understand

Diagnosis	(i) Spend extra time with the patient in case they need assistance to understand what diabetes is and the next steps in management (ii) Explain treatment plan and encourage patient participation

Weight management	(i) Discuss options for a healthy diet and increased exercise if possible (ii) Provide easy-read information on weight management (iii) Find local weight management services suitable for people with IDDs

Social determinants of health	(i) Discuss with the patient or patient's family about potential barriers to access to healthcare such as transportation, cost, and support level (ii) Identify reasonable and achievable goals

**Table 3:** Example flowchart to assist in managing T2DM.

	Baseline	Clinic visit 2	Clinic visit 3	Clinic visit 4	Clinic visit 5
Medical diagnoses					
BMI: (weight/height)					
Blood pressure					
FASTING BG and/or HbA1c					
Lipid profile: (triglycerides/total cholesterol/LDL-C/HDL-C)					
Medication lists (with side effects)					
Physical activity level: (none, low, moderate, and high)					
Description of mood					
Further management					

**Table 4:** Pharmacological treatment for individuals with metabolic syndrome.

Drug class	Mechanism of action	Indication	Side effects	Contraindications	Weight impact
Biguanides (metformin)	(i) Increases peripheral insulin sensitivity and enhances insulin effects (ii) Reduces LDL and increases HDL	Drug of choice in almost all patients with T2DM	(i) Lactic acidosis (ii) GI distress: nausea, vomiting, diarrhea, and abdominal pain (iii) Vitamin B12 deficiency	(i) Renal failure (ii) Severe liver failure (iii) Chronic pancreatitis (iv) Shock (v) Sepsis	Promotes weight loss

SGLT-2 inhibitors (dapagliflozin, and empagliflozin)	(i) Decrease in glucose reabsorption by inhibiting SGLT-2 receptor in the kidney	Treatment-compliant patients with T2DM	(i) Urinary tract infections (ii) Dehydration (iii) Diabetic ketoacidosis	(i) Renal failure (ii) Patients with renal tract abnormalities	Promotes weight loss

GLP-1 receptor agonist (exenatide, liraglutide, and dulaglutide)	(i) Increased insulin secretion (ii) Decreased glucagon secretion (iii) Delayed gastric emptying	Treatment-compliant patients with T2DM	(i) GI distress: nausea and vomiting (ii) Increased risk of pancreatitis	(i) Pre-existing gastrointestinal motility disorders (ii) Chronic pancreatitis (iii) Medullary thyroid cancer or MEN2	Promotes weight loss

DPP-4 inhibitors (sitagliptin, saxagliptin, and linagliptin)	(i) Inhibits degradation of DPP4 (ii) Increased insulin secretion (iii) Decreased glucagon secretion (iv) Delayed gastric emptying	Treatment-compliant patients with T2DM	(i) GI distress: diarrhea and constipation (ii) Increased risk of pancreatitis (iii) Worsening renal function (iv) Headaches, dizziness	(i) Liver failure (ii) Renal failure	No significant weight changes

Thiazolidinediones (pioglitazone and rosiglitazone)	(i) Activation of the transcription factor PPAR*γ* (ii) Increased glucose utilization (iii) Increased fat storage (iv) Decreased hepatic glucose production	T2DM patients with renal failure and/or contraindications to insulin therapy	(i) Increased risk of heart failure (ii) Osteoporosis (iii) Fluid retention	(i) Congestive heart failure (ii) Liver failure	Promotes weight gain

Sulfonylureas (glimepiride, glyburide, and glipizide)	(i) Inhibits ATP-sensitive potassium channels in the pancreas leading to increased insulin secretion (ii) Increased insulin sensitivity (iii) Decreased gluconeogenesis	T2DM patients who have normal BMI and do not consume alcohol	(i) Life-threatening hypoglycemia (ii) Renal failure (iii) Alcohol intolerance (iv) Hemolytic anemia	(i) Beta-blockers (ii) Obesity (iii) Severe liver or renal failure (iv) Sulfonamide allergy (v) Cardiovascular comorbidities	Promotes weight gain

Alpha-glucosidase inhibitors (acarbose and miglitol)	(i) Inhibit alpha glucosidase (ii) Delayed glucose absorption (iii) Decreased carbohydrate breakdown and no risk of hypoglycemia	Treatment-compliant patients with T2DM	(i) GI distress: flatulence, bloating, abdominal pain, and diarrhea	(i) Renal failure (ii) Inflammatory bowel disease	No significant weight changes

Statin (atorvastatin, rosuvastatin, and simvastatin)	(i) Inhibits HMG-CoA reductase enzyme reducing cholesterol synthesis (ii) Upregulates LDL receptors	Treatment of hypercholesterolemia, hyperlipoproteinemia, and hypertriglyceridemia	(i) Myopathy (ii) Rhabdomyolysis (iii) Hepatotoxicity (iv) Increased risk of developing T2DM	(i) Active hepatic disease (ii) Breastfeeding (iii) Other CYP450 inhibitors	Promotes weight gain

Cholesterol absorption inhibitors (ezetimibe)	(i) Inhibit the intestinal absorption of dietary and biliary cholesterol (ii) Lowers LDL-C, modestly decreases triglycerides, and raises HDL-C	Primary hypercholesterolemia	(i) Myopathy (ii) Hepatotoxicity	(i) Hypersensitivity to any component of formulation (ii) Unexplained persistent elevation in serum transaminases	No significant weight changes

SGLT2, sodium-glucose cotransporter 2; GLP-1, glucagon-like peptide 1; DPP-4, dipeptidyl-peptidase 4; LDL, low-density lipoprotein; HDL, high-density lipoprotein; PPAR*γ*, peroxisome proliferator-activated receptor gamma; HMG-CoA, *β*-Hydroxy *β*-methylglutaryl-CoA [75–77].

## Data Availability

No underlying data were collected or produced in this study.
